# Tuberculosis Abscess of the Chest Wall

**DOI:** 10.4269/ajtmh.14-0063

**Published:** 2014-10-01

**Authors:** Juan Cataño, Jefferson Perez

**Affiliations:** Infectious Diseases Section, Internal Medicine Department, University of Antioquia Medical School, Medellín, Colombia; Infectious Diseases Section, Clinica Las Vegas, Medellín, Colombia

## Abstract

A 42-year-old male presented in June of 2011 with nocturnal fevers, night sweats, an 8-kg weight loss, and a cutaneous right chest wall mass. In March of 2013, a computed tomographic scan of the thorax showed a 54 × 18 × 26-mm right lower lobe mass with peripheral calcifications, and in May of 2013, he was admitted for a segmental lobectomy, in which histologic examination of the pulmonary tissue revealed granulomas with multinucleated giant cells. The tissue was negative for acid-fast bacillae on Ziehl–Neelsen stain, and culture grew *Mycobacterium tuberculosis*. Therefore, he was started on four first-line antituberculosis medications and showed rapid symptomatic improvement.

A 42-year-old male patient with an unremarkable past medical history presented in June of 2011 with nocturnal fevers, night sweats, an 8-kg weight loss, and a cutaneous right chest wall mass. He was started on multiple empirical antibiotics for presumptive bacterial infection with no improvement. The mass progressed to two cutaneous chest wall fistulae, at which point additional empirical antibiotics were prescribed with minimal improvement. In March of 2013, he presented to the local infectious disease clinic because of poor appetite, malaise, a 10-kg weight loss, persistent chest wall fistulae, and chest pain. He had normal vital signs, and physical examination was notable only for the presence of two lower right chest wall fistulae (Figure 1A). A computed tomographic scan of the thorax showed a 54 × 18 × 26-mm right lower lobe mass with peripheral calcifications (Figure 1B). In May of 2013, he was admitted for a segmental lobectomy, in which surgeons removed abundant caseous material. Histologic examination of the pulmonary tissue revealed granulomas with multinucleated giant cells. The tissue was negative for acid-fast bacillae on Ziehl–Neelsen stain, and culture grew *Mycobacterium tuberculosis*. A test for human immunodeficiency virus (HIV) was negative, and he was started on four first-line antituberculosis medications. He showed rapid symptomatic improvement and was discharged 2 weeks after admission.

**Figure 1.**
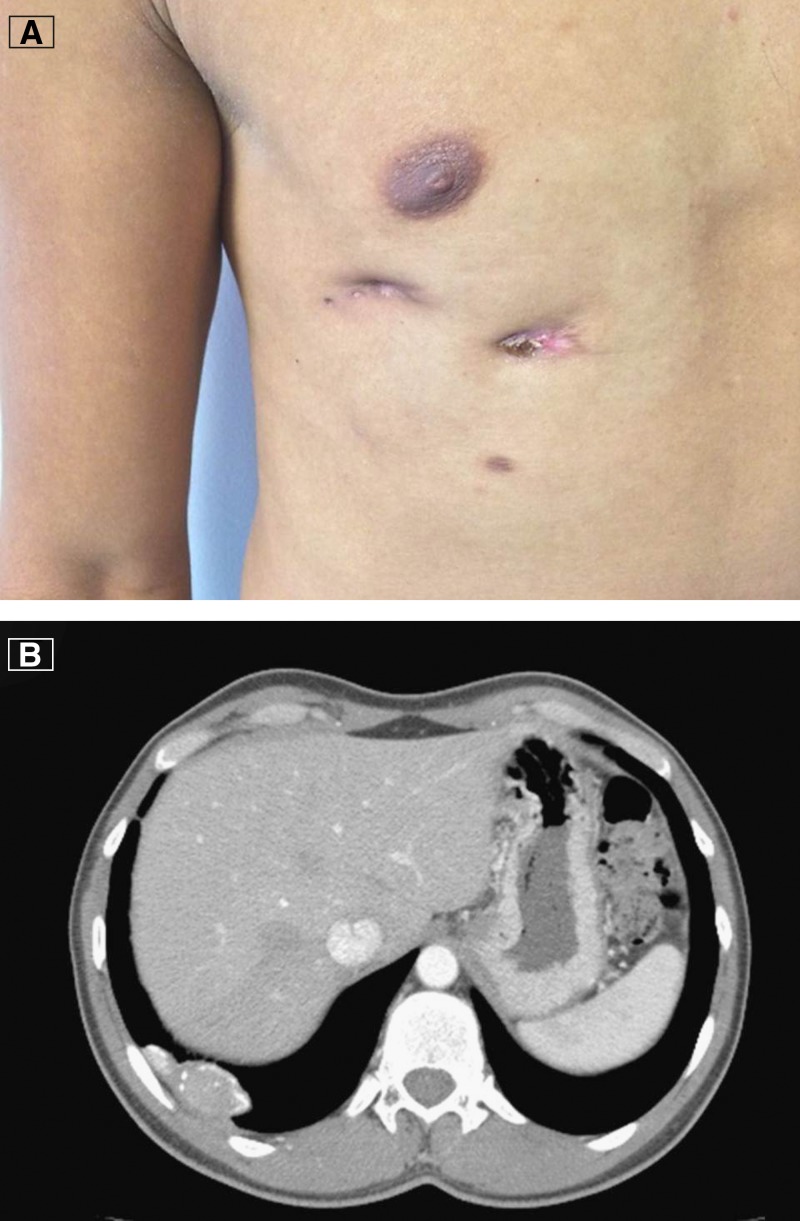
(**A**) Clinical picture of the case. (**B**) Thorax CT scan showing a 54 x 18 x 26-mm right lower lobe mass with peripheral calcifications.
